# The influence of *Akkermansia muciniphila* on intestinal barrier function

**DOI:** 10.1186/s13099-024-00635-7

**Published:** 2024-08-03

**Authors:** Chunyan Mo, Xiran Lou, Jinfang Xue, Zhuange Shi, Yifang Zhao, Fuping Wang, Guobing Chen

**Affiliations:** 1 Medical School, Kunming University of Science and Technology, 727 Jingming South Road, Chenggong District, Kunming, 650500 China; 2 Department of Emergency Medicine, The First People’s Hospital of Yunnan Province, 157 Jinbi Road, Xishan District, Kunming, 650034 China

**Keywords:** *Akkermansia muciniphila*, Intestinal barrier, Inflammation, Immunity, Cross-feeding

## Abstract

Intestinal barriers play a crucial role in human physiology, both in homeostatic and pathological conditions. Disruption of the intestinal barrier is a significant factor in the pathogenesis of gastrointestinal inflammatory diseases, such as inflammatory bowel disease. The profound influence of the gut microbiota on intestinal diseases has sparked considerable interest in manipulating it through dietary interventions, probiotics, and fecal microbiota transplantation as potential approaches to enhance the integrity of the intestinal barrier. Numerous studies have underscored the protective effects of specific microbiota and their associated metabolites. In recent years, an increasing body of research has demonstrated that *Akkermansia muciniphila* (*A. muciniphila*, Am) plays a beneficial role in various diseases, including diabetes, obesity, aging, cancer, and metabolic syndrome. It is gaining popularity as a regulator that influences the intestinal flora and intestinal barrier and is recognized as a ‘new generation of probiotics’. Consequently, it may represent a potential target and promising therapy option for intestinal diseases. This article systematically summarizes the role of Am in the gut. Specifically, we carefully discuss key scientific issues that need resolution in the future regarding beneficial bacteria represented by Am, which may provide insights for the application of drugs targeting Am in clinical treatment.

## Background

**Fig. 1:**
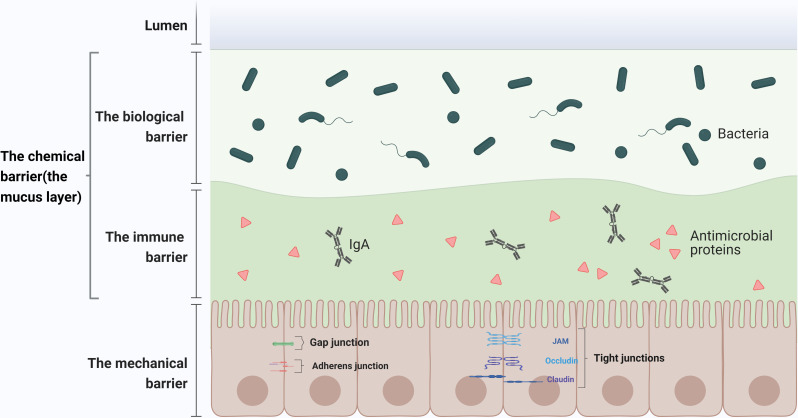
Intestinal barrier structure. The mechanical barrier includes intact epithelial cells, a lipid bilayer with tight brush borders of epithelial cells, and cell junctions at the lateral borders of the cells. The chemical barrier consists of digestive juices secreted by the gastrointestinal tract, digestive enzymes, and antibacterial substances produced by the normal intestinal microbiota. The immune barrier is primarily composed of gut-associated lymphoid tissue and secretory immunoglobulins. The biological barrier represents an interdependent and interacting microecosystem composed of the resident intestinal microbiota

The intestinal barrier structure serves to isolate microorganisms, macromolecular toxins, and other antigenic substances present in the intestinal lumen from the immune cells in the intestinal lamina propria, thereby preventing abnormal activation of the intestinal immune system [1, 2]. The mucosal barrier system produced by intestinal epithelial cells can be categorized into two subtypes: physical and chemical barriers. The biological barrier is comprised of tens of thousands of bacteria residing in the gut. Furthermore, the scattered immune cells, immunoglobulins, and antimicrobial peptides present in the intestine collectively form an immune barrier (Fig. 1). The onset of related diseases can disrupt these intestinal barriers, leading to further disease progression. For instance, in sepsis, a robust and sustained inflammatory response damages the intestinal epithelium, resulting in increased intestinal permeability. This heightened permeability facilitates the entry of endotoxins and opportunistic pathogenic bacteria from the gut into the bloodstream, thus perpetuating a vicious cycle [3, 4]. Additionally, in inflammatory bowel disease (including Crohn’s disease and ulcerative colitis), which is characterized by chronic recurrent inflammation of the gastrointestinal tract, dysregulation of the immune response of the resident microbiota in the intestinal biological barrier plays a pivotal role in mediating the disease. Therefore, the regulation of the intestinal microbiota and the treatment of mucosal inflammation are of paramount importance, alongside conventional therapies such as aminosalicylates, corticosteroids, and immunosuppressants [5, 6].

The human gut harbors trillions of microorganisms, collectively forming a highly intricate gut microecosystem. This ecosystem functions as a biological barrier within the gut, situated within a loose mucus layer. Comprising bacteria, archaea, viruses, and eukaryotes, this microbial community is now recognized as one of the primary factors influencing the regulation of host health. It plays essential roles in regulating host metabolism, maintaining mucosal barrier integrity, and facilitating the maturation and function of the immune system [7]. The dynamic interaction between the microbiota and the host helps to maintain the intestinal epithelial barrier as well as the development and maturation of the host immune system [8]. Changes in intestinal microbiota are closely related to metabolic disorders such as obesity, insulin resistance, glucose intolerance, dyslipidemia, and hepatic steatosis [9]. Chronic low-grade inflammation, changes in the structure or metabolites of the microbiota, and changes in the function of intestinal epithelial cells, intestinal endocrine cells, and intestinal immune cells have been proven or thought to be the causes of metabolic disorders [10–12]. Hence, it may play an indispensable role in the intestinal tract to regulate the composition and function of the intestinal microbiota through direct supplementation of specific bacterial species, drug intervention, or dietary adjustments. In recent years, research on the modulation of the intestinal flora in various diseases has gained momentum, and methods such as fecal microbiota transplantation, probiotics, and prebiotics have gradually gained attention. However, the gut microbiota is a complex system, and determining how to effectively regulate it and identifying the specific gut strains to target are currently challenging issues that require resolution.

Recently, Am has garnered attention from researchers and medical professionals worldwide, emerging as a star in the field. Several studies have highlighted Am’s involvement in regulating host intestinal barrier function and immune response. This leads us to speculate that it may hold significant potential as a probiotic, contributing to human health [13]. Am is an anaerobic, gram-negative, non-motile, and oxygen-tolerant bacterium. It belongs to the phylum *Verrucomicrobia*, class *Verrucomicrobiae*, order *Verrucomicrobiales*, family *Verrucomicrobiaceae*, genus *Akkermansia*, and species *muciniphila*. Currently, the genus *Akkermansia* includes three officially recognized species: *A.muciniphila* [14], *Akkermansia giganiphila* [15], and *Akkermansia biwaensis* [16], which were isolated from human and python feces in 2004, 2016, and 2023, respectively. In recent years, with the development of whole-genome sequencing technology, new species in the genus *Akkermansia* have been gradually discovered, and an increasing number of experimental studies have demonstrated their protective effects in the intestine, such as *Candidatus A.Intenavium*, *Candidatus A. intestinigallinarum* [17], *A.massiliensis* sp. nov [18]. *A.massiliensis*, as the second largest species in the phylum *Verrucomicrobia* after *A.muciniphila*, presents a new opportunity for the development of effective and targeted interventions [19]. Am is among the most common bacteria, constituting approximately 3-5% of the intestinal species in the adult colon and over 1% of the overall fecal microbiota. Its abundance varies with age, diet, body mass, and immune state over a lifetime [20]. Am, specifically the type strain MucT (ATCC BAA-835), was discovered by Derrien et al. in the intestines of both humans and animals in 2004, and has been classified as a new genus in the subfamily *Akkermansia muciniphila* gen., sp. Nov [14]. Like other gut bacteria, Am primarily colonizes the outer mucus layer of the intestine. Its development is sustained by the carbon and nitrogen derived from intestinal mucin, produced by goblet cells to maintain a stable mucus layer, thereby creating a dynamic balance [13, 21, 22]. During this process, carbohydrates are released from the mucin layer, generating organic acids such as acetate and propionate, which contribute to mucus dissolution. Since its discovery, extensive research has established clear connections between Am and metabolic illnesses such as obesity, diabetes, cancer, glucose metabolism, and intestinal functions [23–26]. Its abundance is crucial for healthy physiological functions, and disruptions in its levels can directly impact the pathophysiological progression of chronic diseases [27]. Research indicates that the abundance of Am in the gut is inversely correlated with body weight in both humans and mice. Daily oral administration of Am or its representative membrane protein, AMUC-1100, reverses increased fat mass, adipose tissue inflammation, and insulin resistance in mouse models fed a high-fat diet [25, 28, 29].

Am is also known as the “sentinel of the gut”, has been demonstrated to enhance the integrity of the gut barrier, regulate the immune reaction, lessen inflammatory response, and support the reproduction of butyrate-producing bacteria. The positive benefits of Am have been linked to certain cell components or chemical compounds it produce, including Amuc_1100 [30], Amuc_2109 [31], Amuc_2172 [32], Protein 9 (P9) [33],, and diacyl phosphatidylethanolamine (PE) [34]. With its unique function and widespread presence across almost all life stages, Am has opened up new possibilities for its utilization as next-generation therapeutic probiotics. To offer fresh perspectives for Am research, this review aims to elucidate the protective mechanisms of this organism in maintaining the functioning of the intestinal barrier.

### Effects of Am on intestinal barrier

#### Enhancement of intestinal barrier integrity

The mucus layer serves as the initial physical barrier in the intestine. It covers the intestinal epithelial cells and constitutes an integral structural component of the mammalian intestine. Its primary function is to safeguard the intestinal epithelial cells from damage caused by food and digestive secretions [35]. Mucins are translated and synthesized in the rough endoplasmic reticulum of secretory cells, such as goblet cells. Upon release from goblet cells, the polymerized MUC2 anchors to intestinal epithelial cells, forming the inner mucosal layer. Subsequently, the polymerized MUC2 undergoes hydrolysis by either the host or bacteria, transforming into the outer mucosal layer. This process results in the formation of a mucous bilayer comprising a firm inner layer and a loose outer layer. While the outer mucus layer facilitates colonization by intestinal microbiota, the polymerized inner layer of mucus acts as a barrier, preventing microorganisms from easily invading colonic epithelial cells [36, 37]. If the mucus layer breaks down, pathogenic bacteria can directly contact intestinal epithelial cells, triggering an inflammatory immune response. The integrity of the intestinal barrier is crucially maintained by tight junctions with intestinal epithelial cells, along with the contribution of the mucus layer to the barrier. In various pathological conditions such as infection or dysbiosis, the permeability of intestinal epithelial cells may be significantly increased, leading to a condition known as “leaky gut“ [38, 39]. There are molecules known to transiently disrupt the tight junction proteins (TJs) between epithelial cells, with Zonulin being the sole identified and described molecule thus far. Zonulin, a multidomain protein, is produced into the lumen by mammalian enterocytes in response to stimulation by gut pathogens and gluten. It acts as a molecular switch, leading to the deconstruction of TJs and increasing epithelial permeability [40].

### Am increases the number of goblet cells and mucus secretion

According to earlier studies [28], Am colonization can enhance the population of mucus-producing goblet cells in the colon. Additionally, there is a positive correlation between the abundance of Am and the amount of mucins in the cecum of rats on diets with or without prebiotics [41]. In a study of the host and gut microbiota, Org et al. found that treatment with Am enhanced the number of goblet cells and mucus production in the stomach [42]. Furthermore, in metabolic diseases or DSS-induced colitis, Am may increase goblet cell mucin production and restore gut barrier function [43]. A recent study has shown that antibiotic therapy decreased the quantity of Am but also reduced mucolytic activity and the expression of the gene MUC2, which encodes the main mucin of the colonic mucus [44].

**Fig. 2:**
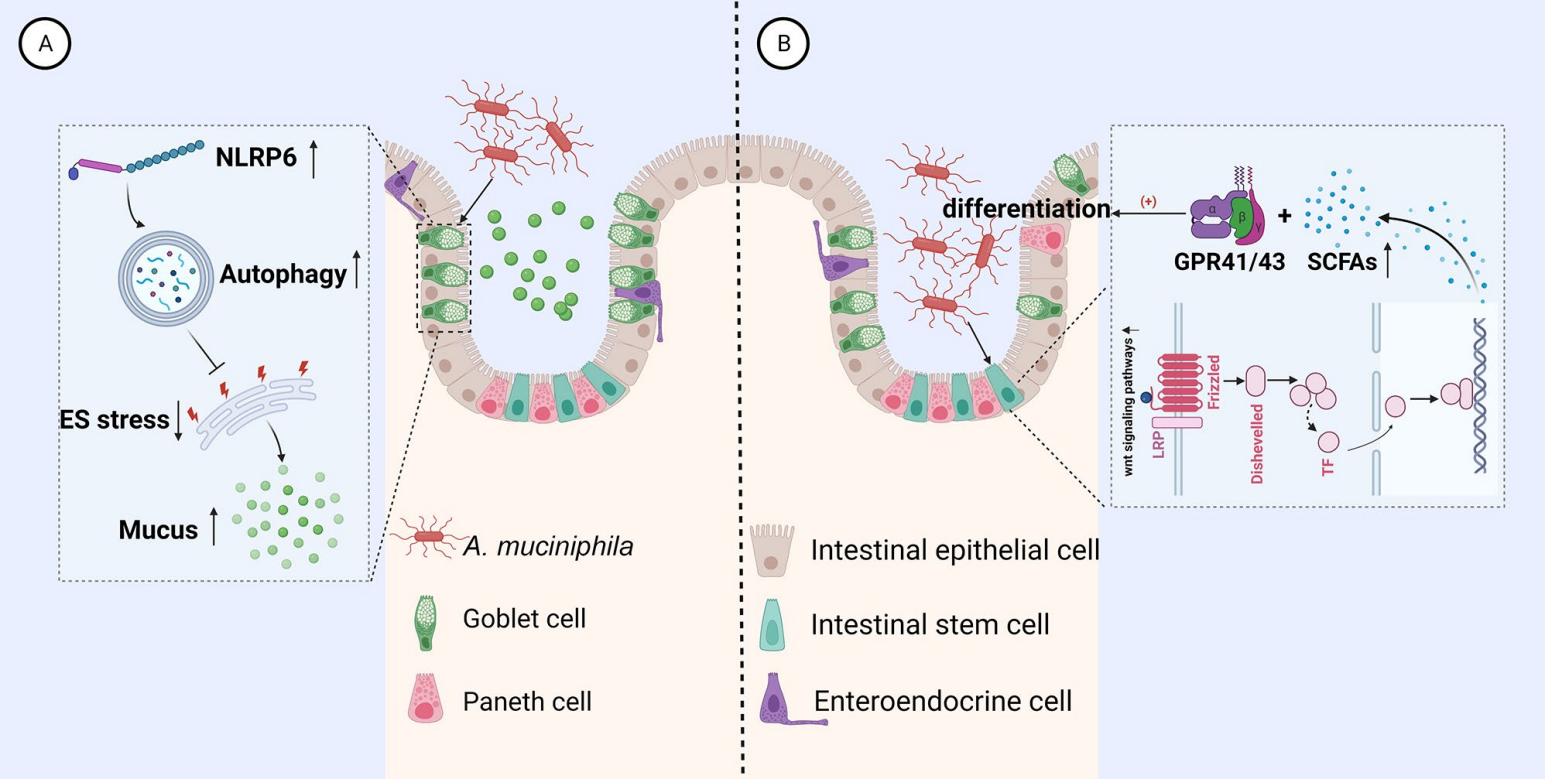
The mechanism by which Am increases intestinal integrity. (**A**): Am upregulates the expression of the NLRP6 inflammasome, which accelerates autophagy in goblet cells and reduces endoplasmic reticulum stress, thereby promoting mucus secretion. (**B**): Am increases the expression of the Wnt signaling pathway and promotes the production of SCFAs, which interact with GPCR41/43 to maintain the proliferation of ISCs and promote the differentiation of Paneth cells and goblet cells

Autophagy has been identified as essential for goblet cells to maintain appropriate secretory activity, as per earlier mechanistic studies [45]. According to Yu et al., Am colonization up-regulated the expression of the NLRP6 inflammasome and accelerated autophagy in goblet cells, thereby increasing the production of fresh mucins that could potentially protect the intestinal mucosal barrier [46]. In-depth research by Naama et al. revealed that endoplasmic reticulum (ER) stress serves as an intrinsic switch regulating goblet cell mucus output, and autophagy may modulate mucus secretion by reducing ER stress, thereby preserving intestinal homeostasis [45] (Fig. 2). However, it remains unknown whether this relationship is directly regulated by Am itself, its metabolites, or by interactions with specific microbiota. Contrary to popular belief, researchers have demonstrated that Am stimulates the growth of mucin-secreting goblet cells, a process previously diminished by a high-fat diet. Further research is required to fully elucidate these mechanisms.

### Am accelerates intestinal epithelial cells (IECs) development

Intestinal barrier development and goblet cell proliferation are propelled by the proliferation of intestinal stem cells(ISCs). The proliferation and differentiation of these ISCs facilitate the regeneration of IECs and repair the damaged intestinal mucosal barrier. Both IECs and goblet cells originate from stem cells located at the base of the intestinal crypt [47]. The maintenance of stem cells and the differentiation of intestinal cell lineages involve a variety of complex signaling pathways, including the Wnt/β-catenin, Notch, and BMP signaling pathways [48]. The gut microbiota and the metabolites it produces can exert various effects on epithelial cells and may serve as the missing link connecting the effects of diet on the microbiota to mucus properties [49]. The bacterial metabolites involved in these interactions are highly diverse, encompassing a range of molecules from small to large. These include by-products of bacterial metabolism, such as short-chain fatty acids (SCFAs) and bile acids (BAs), among others, as well as complex macromolecules essential for bacterial integrity, such as peptidoglycan and lipopolysaccharide (LPS) [50]. These metabolites, such as BAs, play a role in regulating the production of mucus in gut goblet cells. BAs have been demonstrated to support barrier function by inducing mucus secretion by goblet cells, promoting epithelial cell migration, and enhancing intestinal innate immune defenses [51].

In addition to metabolites, various microbial communities in the gut have been found to play an important role in the growth and development of normal epithelial cells and the recovery of damaged intestinal mucosa [31, 32]. In a study of spaceflight-related metabolic disorders, the authors found that regular oral administration of *Bifidobacterium spp*. to mice improved intestinal epithelial homeostasis and decreased intestinal permeability [52]. Am has previously been found to have a tight relationship with epithelial surfaces [22] and to have an impact on the expression of epithelial genes [21]. Additionally, it was discovered in Alam’s study that Am can facilitate mucosal wound healing through FPR1-dependent redox-mediated epithelial proliferation and migration regulation [53].

Kim et al. discovered that the strain of Am from healthy human feces is superior to commercial strain, for it may enhance the proliferation and differentiation of ISCs. Previous studies have demonstrated that the Wnt signaling pathway is crucial for fostering and sustaining ISC and IEC differentiation proliferative activity [54, 55]. On the basis of this, they discovered that this function is directly tied to SCFAs, which may supply the IECs with 70% of their energy fuel in order to preserve the integrity of the intestinal barrier [56]. The specifics are that Am treatment increased the expression of Wnt signaling pathway genes and production of SCFA metabolites, such as acetic and propionic acids, which interact with G protein-coupled receptor (GPCR) 41/43 and, in turn, maintain the stemness of ISCs, promoting the differentiation of paneth cells and goblet cells [57](Figure 2). Additionally, their recent study found out Am treatment may elevate the production of lipid metabolites, including myristic and palmitic acid, which influence IEC development [58]. In the latest study, Kang et al. discovered that the newly identified secreted protein Amuc_1409 dissociates β-catenin from E-cadherin by interacting with the EC domain of E-cadherin, thereby activating the Wnt/β-catenin signaling pathway and promoting the regeneration of ISCs [59]. Furthermore, other research showed that the amount of Am is positively correlated with the activity of the epithelial tryptophan metabolizing enzyme indoleamine 2,3-dioxygenase 1 (IDO1). IDO1 enhances markers of stem, goblet, Paneth, enteroendocrine, and tuft cells via interaction with the Aryl Hydrocarbon receptor. The ability of Am to control IDO1 production directly has not yet been demonstrated. In order to rule out this option, more research should be conducted [60]. In addition to Am, the role of new species in the phylum *Verrucomicrobia* in the intestinal barrier is gradually being discovered. In 2024, Huang et al. isolated a new species, *Akkermansia sp. BCRC 18,949*, from the feces of healthy Taiwanese individuals and found that it has enhanced protective effects against DSS-induced colitis [61].

### Am upregulates the expression of tight junction (TJ) proteins

The key regulators of intestinal epithelial TJ barrier function are the TJ proteins [62]. TJs consist of transmembrane proteins (e.g., occludin and claudin), peripheral membrane proteins (e.g., ZO-1 and ZO-2), and regulatory proteins. TJ proteins control the passage of molecules through the epithelial layer based on their size and charge [63]. They are crucial for maintaining a robust epithelial barrier and gut health. These cellular junctions physically impede the invasion of microorganisms through the paracellular pathway [64].

According to certain investigations, Am might enhance gut barrier function by upregulating the expression of TJ genes. Additionally, it has been demonstrated that Am extracellular vesicles (AmEVs), which have been suggested to mimic some of the positive effects of the bacteria, may reduce intestinal permeability in mice by modulating tight junctions [65–67]. Liu et al. discovered the potential impact of Am and pasteurized Am on the genetic expression of ZO-1, Occludin, and Claudin3, which are associated with gut barrier function, in a rat experiment involving *Salmonella Typhimurium* infection [68].

Although studies indicate that Am enhances the function of the epithelial barrier by regulating TJ proteins, the precise mechanisms by which the bacteria achieve this positive impact are not fully understood. Investigations into the processes through which Am controls TJ proteins are relatively limited. Previous research has demonstrated that the activation of several signaling pathways, including the NF-κB pathway, underlies the damage to intestinal TJ barrier function caused by the pro-inflammatory cytokine IL-1 [69]. Through the inhibition of the NF-κB signaling pathway, Pan et al. and colleagues consistently demonstrated that Am restored the production of TJ proteins and protected the epithelial barrier from IL-1-induced damage [67]. Further experimental research is needed in the future to confirm whether other pathways are involved in this process, which remains unclear at this time.

## Inhibiting intestinal inflammation

Epithelial barrier dysfunction, often referred to as “leaky gut” syndrome, commonly coexists with intestinal inflammation. The positive effects of Am on gut barrier function in the context of intestinal inflammation have been documented, along with alterations in gut permeability associated with metabolic diseases. One study demonstrates that Am can mitigate intestinal inflammation induced by E. coli by suppressing the production of inflammatory molecules such as TNF-α and IL-8 [67]. Additionally, compared to healthy individuals, the prevalence of Am was significantly lower in patients with Crohn’s disease and ulcerative colitis [70, 71]. This link has been causally examined in preclinical models. Initially, Am has been shown to exert positive effects on colitis, with AmEVs protecting mice from developing dextran sulfate sodium (DSS)-induced colitis [72]. Subsequent investigations found that Am improved DSS-induced colitis and restored gut barrier function while reducing weight loss, colon length shortening, and histopathological scores [73].

### Am could stimulate L cells to secrete glucagon-like peptide 1 (GLP-1) through various pathways, thereby suppressing levels of intestinal inflammation

Enteroendocrine L cells secrete the hormone GLP-1, which was initially classified as a glucoregulatory hormone but also demonstrates anti-inflammatory properties and supports mucosal integrity [74]. In Lebrun’s study, it was observed that circulating GLP-1 levels sharply increased during ischemia/reperfusion trials in mice or experimental disruptions of gut barrier integrity following dextran sodium sulfate administration. Moreover, this phenomenon was detected prior to noticeable changes in inflammatory state, plasma cytokine levels, or LPS levels. LPS treatment also stimulated GLP-1 secretion in human subjects. Furthermore, they demonstrated that LPS injection in mice led to a rapid increase in plasma GLP-1 levels via a Toll-like receptor 4 (TLR4)-dependent mechanism. These findings connect glucagon-like peptide secretion to gut inflammation, expanding traditional understandings of enteroendocrine L cell function to include the perception of inflammatory stimuli and compromised mucosal integrity. Am is also closely associated with GLP-1, given its role as a crucial mucolytic bacterium in the colon. Am has been shown to induce intestinal L cells to release GLP-1 in several studies, with recent investigations identifying various potential biomolecules as contributing factors [33, 75].

**Fig. 3:**
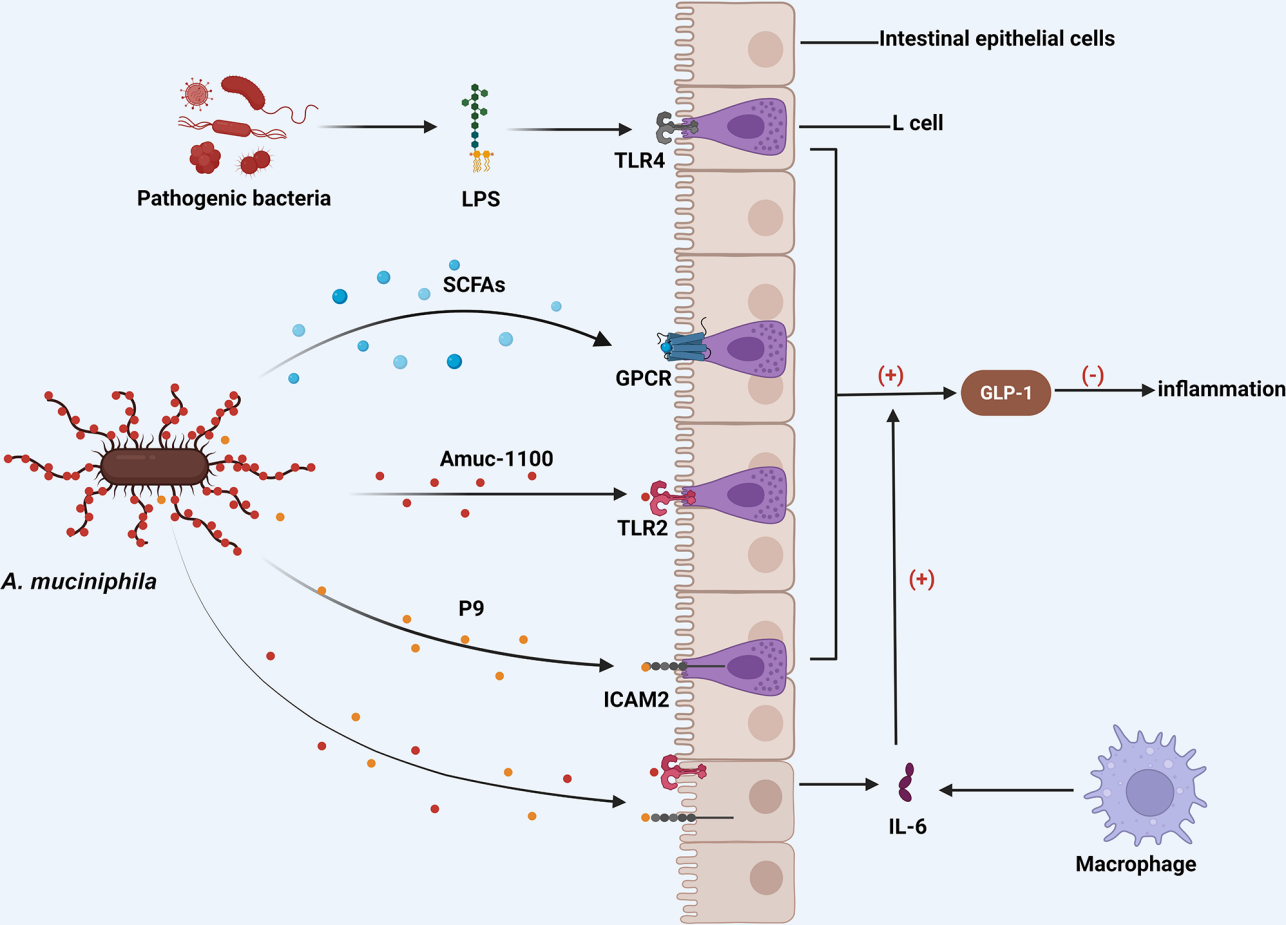
Am promotes the secretion of GLP-1. The surface membrane proteins of Am and its metabolites, SCFAs, can interact with various receptors (GPCRs, TLR2, ICAM2) on IECs to directly or indirectly increase the production of GLP-1, which inhibits the inflammatory response

Am’s mechanism of action is closely linked to SCFAs, as mentioned earlier. According to reported studies, SCFAs can modulate inflammation induced by leukocytes in various disorders by binding to histone deacetylase (HDAC), GPR41, GPR43, and GPR109A receptors [76]. Similar to how Am degrades mucus, acetate and propionate are known to bind to GPR41/43 receptors expressed on L cells, thereby increasing the production of GLP-1 while reducing inflammation levels. Butyrate also regulates GLP-1 synthesis and the development of immune cells, inhibits inflammatory factors such as IL-8 and IL-1, and promotes the production of anti-inflammatory factors like IL-10. Additionally, it exerts anti-inflammatory effects by activating GPR109A receptors [77, 78]. Surprisingly, the anti-inflammatory properties of butyrate have also been associated with the NF-κB pathway and the generation of anti-inflammatory cytokines by neutrophils and monocytes [79, 80]. However, it remains unclear whether this impact is specifically attributable to Am (Fig. 3).

Its surface membrane proteins, as well as its metabolites, are implicated in Am’s beneficial effects. A unique protein named Amuc_1100 was identified in the outer membrane of Am in 2017 by Plovier et al. Furthermore, it was demonstrated that Amuc_1100 selectively activates TLR2 and binds to L cells to promote GLP-1 secretion to some extent. Most of Am’s positive effects have been observed in disease models of intestinal inflammation and colon cancer with Am administration alone [24, 30, 81]. Importantly, many researchers, including them, found that the protein also retains its activity even when exposed to pasteurization temperatures [82]. However, a few researchers have observed that inactivated Am loses its ability to reduce inflammation in mouse models [83, 84] (Fig. 3).

In a 2021 study, another protein named P9, encoded by the Amuc_1831 gene previously discovered, was identified. Unlike Amuc_1100, which is a component of Am, P9 is secreted by this organism. It’s noteworthy that P9 directly stimulates L cells to release GLP-1 by binding to the intercellular adhesion molecule 2. Additionally, it can increase the release of IL-6 by intestinal epithelial cells and/or macrophages, indirectly promoting the secretion of GLP-1, although the mechanism remains unknown [33](Figure 3). Subsequently, several scientists have raised questions about this protein. For example, how does it influence the gut’s L cells to promote the release of the hormone? Does Am affect physiological conditions through P9? Does P9 exclusively exist in Am, or do other bacterial species also express and produce it [85]? Furthermore, what impact does P9 have on the ability of L cells to release other gut peptides like GLP-2 and Peptide YY [86] ?

### Other ways of Am reducing inflammation in the intestinal barrier

One of the most crucial members of the NLR family of pattern recognition receptors is the Nucleotide Binding Oligomerization Domain-Like Receptor Family Pyrin Domain-Containing 3 (NLRP3). This receptor can identify pathogens inside cells as well as danger signals emanating from the cells. Upon recognition, it leads to the formation of a protein complex known as an “inflammasome,” comprising the linker protein ASC and pro-caspase-1 [87]. The NLRP3 inflammasome is abnormally associated with a number of diseases [88]. According to research, Am can activate NLRP3 to treat acute colitis brought on by DSS [89]. By lowering intestinal inflammation and enhancing the expression of tight junction protein, Qian et al. discovered that the release of -acetylaminohexosidase (Amuc_2109) by Am can also mitigate DSS-induced colitis in mice [31]. Pasteurized Am has the identical anti-inflammatory properties, according to other studies [68]. The mechanism may be that Am increases M2 macrophage polarization while also producing more reactive oxygen species (ROS), nitric oxide (NO), and inflammatory cytokines to encourage NLRP3 expression and boost macrophage antimicrobial activity [68, 90]. To corroborate the above mentioned mechanism, more experimental or clinical researches are required. It is unknown, though, if other modes of action are involved.

BAs can activate a variety of signaling pathways as metabolic integrators and signaling factors, with farnesoid X receptor (FXR) and G protein-coupled bile acid receptor 1 (GPBAR1/TGR5) being the most often described ones [91]. BAs interact with TGR5 to prevent hepatocyte lipid peroxidation, increase energy expenditure, and control immunity [92], but nothing is known about how they affect the intestinal barrier. Serum and stool samples from individuals with the severe fever with thrombocytopenia syndrome were examined by Xie et al. They discovered that whereas Am was more prevalent in the intestines of infected individuals, it was less prevalent in the intestines of dead patients. They also discovered a myxotropic-BA-TGR5 axis that inhibits host NF-κB-mediated immunopathogenic reactions brought on by Severe Fever with Thrombocytopenia Syndrome Virus infection and perhaps other systemic viral pathogens [93].

The NF-κB pathway plays a crucial role in cellular responses to external stimuli and is essential for cellular inflammatory and immunological reactions. Similarly, Am interacts with TLR2 and TLR4 receptors to activate the NF-κB pathway, which in turn stimulates peripheral blood mononuclear cells to produce IL-1, IL-6, IL-8, IL-10, and TNF-α. This pathway not only regulates the intestinal immunological microenvironment but also helps prevent intestinal inflammation. The LPS from Am, the purified outer membrane pilus-like protein Amuc_1100, or the extracellular vesicles (EV) generated by Am may all play a role in how the organism interacts with TLR2/4 [72].

While some research has indicated that Am is beneficial as an anti-inflammatory probiotic, others have suggested that this bacterium actually exacerbates gut inflammation by degrading mucin. According to studies, individuals with inflammatory bowel disease exhibit an increase in the overall number of bacteria associated with the mucosa [94]. Researchers propose that this phenomenon occurs because Am compromises the integrity of the mucosal layer, making it easier for luminal bacteria and antigens to penetrate intestinal epithelial cells and the immune system, thereby potentially causing or exacerbating inflammatory diseases [95–97] and even being linked to specific types of cancer [98]. However, due to the limited number of mucin degraders and associated enzymes that have been studied, the systematic role of mucin-degraders in intestinal homeostasis and dysbiosis has not yet been fully elucidated [99].

## Regulating the intestinal immune response

Due to the high bacterial density, our intestines serve as a unique immunological site where host-microbiota interactions occur. The gut immune system balances immunity to pathogenic bacteria with tolerance to commensal bacteria to maintain a controlled response to commensal bacteria under normal conditions. Immunosuppressive mechanisms are therefore crucial for maintaining intestinal homeostasis. In recent years, Am, a well-known symbiotic bacterium in the intestine, has been found to modulate gut immunity. Several studies have demonstrated a correlation between immunological disorders such as asthma, atopic dermatitis, and the abundance of Am in the intestine [100]. Am has also been demonstrated to influence host intestinal immunity in several researches on intestinal illnesses, such as IBD, colitis, colon cancer, and so forth [101, 102]. Although the mechanisms by which Am induces these immune responses are still poorly understood, some are currently being investigated.

### Am regulates immune cells functions

Numerous experimental investigations have demonstrated that Am and Amuc_1100 play crucial roles in immune regulation and hold promising potential in immunotherapy due to their prevalent presence on the “beneficial” side. Consequently, there is increasing clarity on how Am and its membrane proteins contribute to the initiation and progression of intestinal cancer. In a study by Routy B. et al., the gut microbiome of 100 patients with non-small cell lung cancer and renal cell cancer who responded effectively to anti-PD1 antibodies was thoroughly analyzed, revealing enrichment in Am [103].

**Fig. 4:**
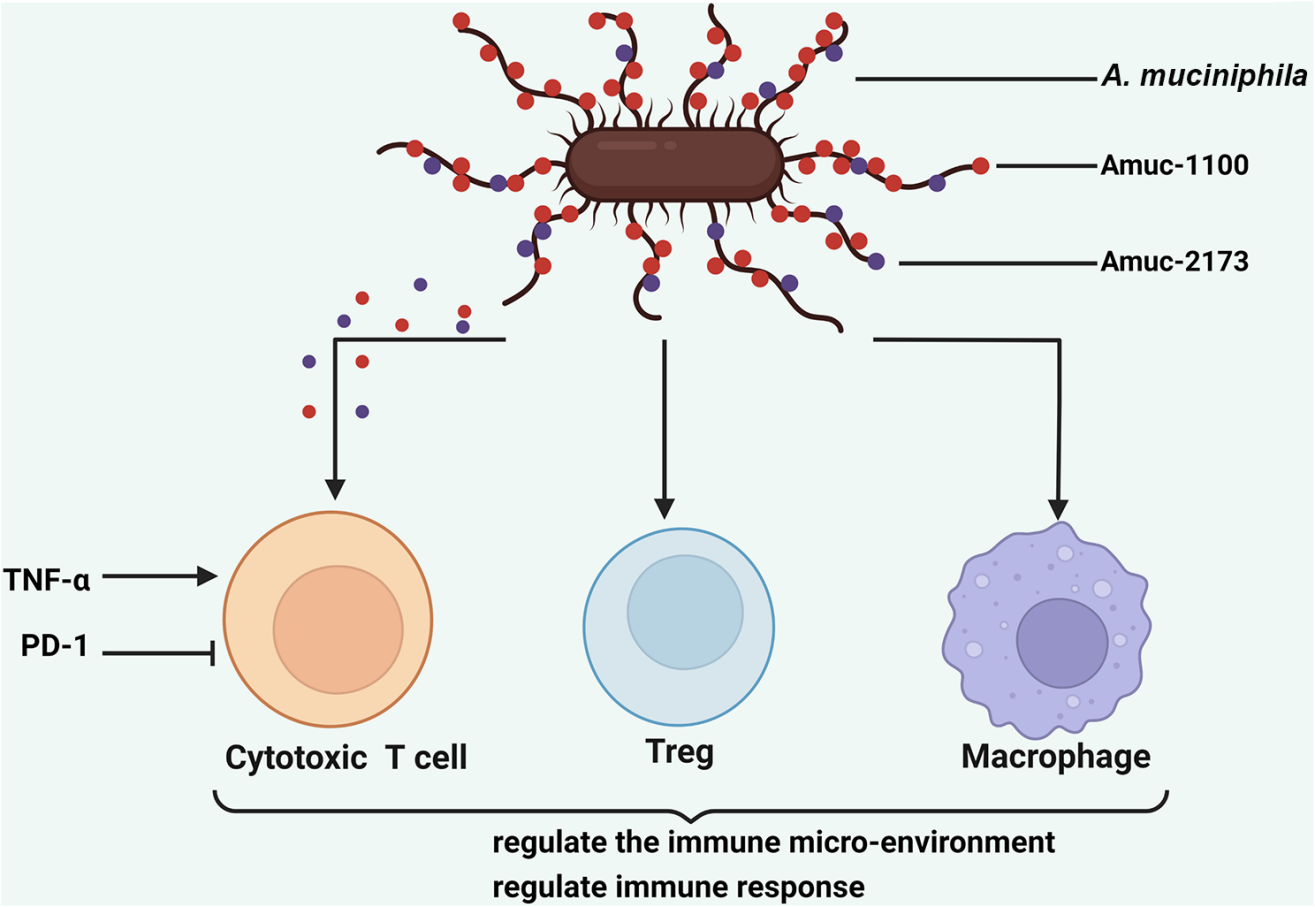
Am regulates Immune environment. The surface membrane proteins of Am can enhance the immune microenvironment by modulating CTLs, promoting T cell differentiation, and inducing macrophage activation

Am and Amuc_1100 have been shown to inhibit colitis-associated colorectal cancer by increasing and activating CD8 + cytotoxic T lymphocytes (CTLs), leading to TNF induction and PD-1 down-regulation. Surprisingly, other studies have found that inactivated Am and Amuc_1100 exhibit similar effectiveness [24]. In 2023, researchers discovered Amuc_2173, a distinct membrane protein present on the surface of Am, which regulates CTLs to control the microenvironment of intestinal tumors. Unexpectedly, Amuc_2172 was found capable of entering colorectal cells via macropinocytosis and acting as an enzyme that acetylates Lys14 on histone H3, thereby altering the protein’s post-translational modification in host cells and enhancing the immunological microenvironment [32]. Am also interacts with TLR4 to modify the RoR-T + regulatory T cell immunological response, as shown by Liu et al. [102]. Furthermore, research found that naive CD4 + CD44-Foxp3-T cells can be converted to the Treg lineage by microbial antigens from Am, thus becoming anergic [104]. Am can influence not only T cells but also macrophages. Research conducted by Fan’s team on colorectal carcinogenesis demonstrated that Am induced M1-like macrophage activation both in vivo and in vitro. This effect was mechanistically mediated by TLR2/NLRP3-dependent signaling [105] (Fig. 4). These studies provide critical insights into tumor immunotherapy and novel ideas for secure medication delivery techniques. While further research is needed to gain a deeper understanding of the mechanism of action, strain specificity, and the necessity for live cells, human clinical trials are essential to demonstrate the contribution of Am to enhancing immunotherapies.

### The metabolites of am possibly play a part in intestinal immunity

While other anaerobic bacteria encourage the synthesis of butyric acid, Am breaks down mucins to create acetic acid and propionic acid. Some researchers argue that SCFAs are crucial regulators of the immune system due to the extensive expression of SCFA receptors in immune cells [77]. The impact of SCFAs on T cell development into both effector and regulatory T cells has recently been revealed, associated with either immunity or immune tolerance, depending on the immunological milieu [106], and mounting data supports the unique involvement of SCFAs on Treg cells. Recent research has demonstrated that SCFAs can increase host immune tolerance to commensals through interactions with their host’s GPCR and/or HDAC targets, an increase in IL-18 IECs, and suppression of Treg itself or DC expansion/differentiation [107–109]. Additionally, SCFAs can influence the immune system’s ability to identify and destroy pathogens by triggering the activities of effector T cells, which are also known to be controlled by SCFA-mediated HDAC inhibition [108]. Similarly, several investigations have demonstrated that Am can control immunity by regulating Treg cell differentiation. However, the direct relationship between Am, SCFAs, and GPCR/HDAC has not yet been verified in additional research.

## Modulating the endocannabinoid (eCB) system

The eCB system is an endogenous signaling pathway comprising Cannabinoid receptors (CBRs), their corresponding ligands (endocannabinoids), and the enzymes responsible for their production and degradation [110]. The majority of endocannabinoid receptors are CB1 and CB2 receptors, both of which are seven transmembrane GPCRs. Other endocannabinoid receptors include peroxisome proliferators-activated receptors (PPARs), transient receptor potential ion channels (TRPs), GPCR55, and serotonin 1 receptor [111]. The eCB system was found to significantly influence the control of gut barrier function in 2010 [112]. Interestingly, alterations in the gut microbiome were linked to the function of this eCB system tone. It has been shown that some gut microorganisms, such as Am, can adjust the activity of the eCB system [113].

**Fig. 5:**
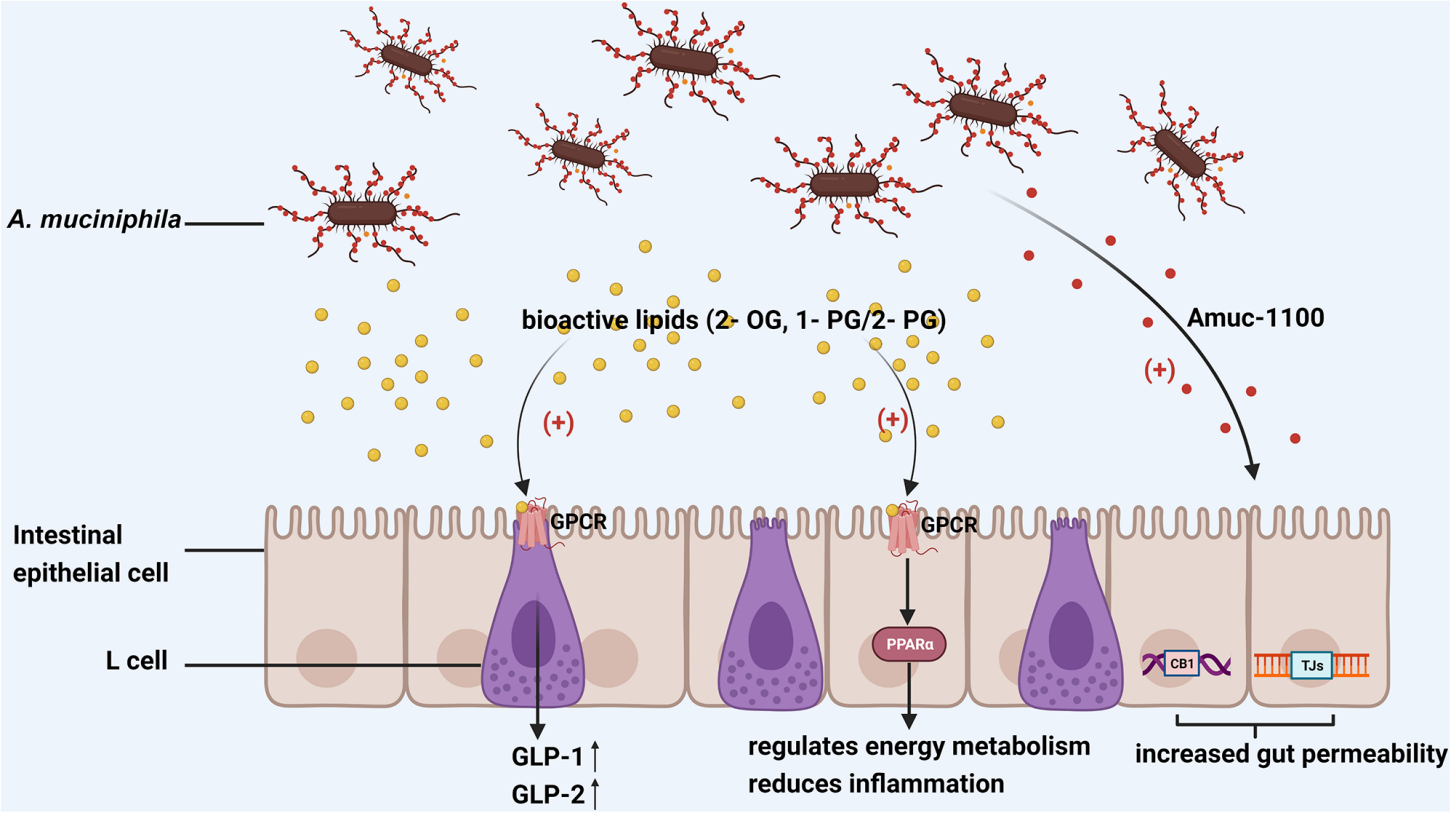
eCB systems are also affected by Am. Am stimulates the IECs of the eCB system to produce 2-OG and 1-PG/2-PG, ultimately promoting the production of GLP-1 and/or GLP-2. It also activates PPARs to promote fatty acid oxidation, control energy metabolism, and reduce inflammation. Additionally, Amuc-1100 reduces intestinal permeability by upregulating the expression of genes encoding tight junctions while downregulating CB1 receptor

In Depommier et al.‘s discovery, it was demonstrated that Am stimulated the production of specific bioactive lipids (2-OG, 1-PG/2-PG) by the IECs from the eCB system, ultimately contributing to the stimulation of GLP-1 and/or GLP-2 and activating the nuclear receptor PPAR, which is expressed throughout the mammalian gut [114]. In turn, PPAR activation encourages fatty acid oxidation, controls energy metabolism, lowers inflammation, and is crucial for maintaining intestinal function [115]. Additionally, PPAR activation may help dendritic cells continue to exhibit tolerogenic behavior against antigens in the diet or commensal microbiome [116]. Meanwhile, Amuc-1100, a surface membrane protein of Am, helps to strengthen the intestinal epithelial barrier by up-regulating the TJ genes, encoding claudin3 and occludin, and simultaneously down-regulating the cannabinoid receptor CB1, which has previously been linked to increased gut permeability [30] (Fig. 5). Fenofibrate and Wy-16,434, two PPAR agonists that already exist, lengthen the small intestine’s villi but do not deepen the crypts [117]. Further investigation is required to ascertain whether Am modulates the eCB system through other chemicals or pathways.

## Cross-feeding other microbiota

The stability and performance of the microbial community depend on “cross-feeding”, or the metabolic interactions between bacteria inhabiting the same topologic niches when nutrients are scarce [118–120]. Anaerobic bacteria in the gut microbiota have a crucial interaction that influences their metabolic pathways and promotes their development. Within the intestinal mucosa environment, several anaerobic bacteria also play significant roles. The term “non-mucodegrading butyrate-producing bacteria” refers to anaerobic bacteria that can only grow and produce butyrate when provided with carbon and nitrogen sources that have already been broken down by mucin-degrading bacteria. Am, a classic example of mucin-degrading bacteria, naturally contributes significantly to the cross-feeding within the intestinal mucus layer.

**Fig. 6:**
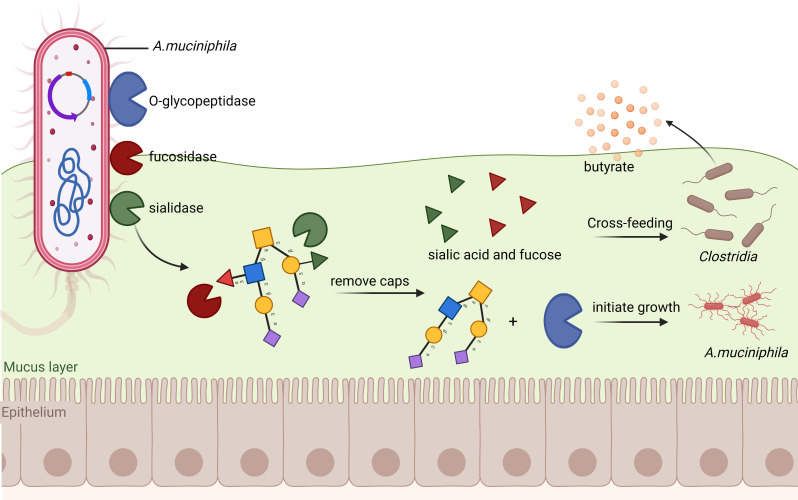
Cross-feeding between Am and *Clostridia*. In the Am, fucosidase and sialidase eliminate all recognized sialic acid and fucosylmucin caps while generating locations for O-glycopeptidase to initiate Am development. The sialic acid and fucose produced during this process are cross-fed to *Clostridium* to form butyrate

Following the breakdown of mucin, Am generates acetate, propionate, and monosaccharides derived from mucin. These compounds serve as substrates for butyrate-producing bacteria that do not degrade mucin [121]. The study by Elzinga et al. showed that the binding of Am to mucin is dependent on preferential LacNAc epitopes carried on O-glycans, which may be exposed upon sialylation by endogenous neuraminidase enzymes such as Amuc_0625 and Amuc_1835 [122]. Pichler et al. discovered that co-culture of Am with *Clostridiales* increases the human milk oligosaccharides pathways, which boost the growth of *Roseburia* and *Eubacterium* while cross-feeding on mucin in their investigation of the early life human gut microbiota [123]. Their attention is then drawn to how weaning-triggered microbiota maturation is impacted, while the significance of glycans and Am is not sufficiently discussed. *Bifidobacterium* bifidum extracellular fucosidases and sialidases were discovered to mediate cross-feeding on mucin with other infant gut *bifidobacteria* in another research of newborns [124]. Interestingly, adults exhibit the same behavior. In several Am strains, Shuoker’s team discovered a significant frequency of the genes encoding the most active fucosidase and sialidase, highlighting the significance of these enzymes for digesting mucin. Additionally, they discovered that these enzymes eliminate all recognized sialyl and fucosyl mucin caps while generating locations for Am O-glycopeptidases to allow cleavage of the mucin backbone and set off the Am development. Surprisingly, the sialic acid and fucose generated during this process do not support the growth of Am but instead encourage the formation of butyrate by co-cultured *Clostridia*, a sialic acid-using model butyrogenic organism [125] (Fig. 6). Their research provides previously unheard-of molecular insight into how Am initiates the degradation of mucin O-glycan and how mucus-associated bacteria share nutrients. However, further research is required to understand the subsequent processes involved in Am’s degradation of mucin. More importantly, it remains unknown whether Am interacts with intestinal anaerobic microorganisms other than *Clostridia*.

## Prospect

Our review comprehensively summarizes the role of Am in the intestinal barrier and elucidates its research mechanisms from four perspectives: intestinal integrity, inflammation, immunity, and cross-feeding. Am, as a prototypical representative of mucin-degrading bacteria in the intestine, exerts influence on various organs and disorders throughout the body. It is imperative to underscore that Am exhibits pleiotropic physiological effects, impacting energy production, lipid and glucose metabolism, inflammation, immunity, brain function, among others. Nevertheless, its significance in intestinal diseases remains contentious, with numerous unanswered questions. Some studies suggest that Am may exacerbate inflammatory responses, while inactivated Am shows no such effect.

To substantiate the plethora of positive effects observed in animal models, further fundamental experimental studies and additional human trials are warranted to ascertain the efficacy of Am in treating intestinal diseases. Moreover, optimal therapeutic dosage and administration routes require exploration. The structure of Am, particularly in relation to Amuc_1100 and other produced compounds (such as extracellular vesicles), warrants further investigation. Beyond the membrane proteins mentioned, additional proteins or compounds within Am may be implicated in its activity, facilitating the identification of potential therapeutic targets.

Advancements in omics technologies, such as metabolomics and genomics, hold promise in addressing these unresolved issues. Since its isolation in 2004, Am MucT has consistently demonstrated positive benefits in the majority of investigations. However, further analysis of strains beyond Am MucT is necessary. Challenges in mass production arise due to Am’s anaerobic nature and its dependence on mucin in the intestinal mucus layer, complicating its application in clinical settings. Addressing these challenges necessitates improvements in microbiological culture techniques and staff expertise, alongside a comprehensive exploration of Am’s mechanism.

## Data Availability

No datasets were generated or analysed during the current study.
